# Development and Assessment of an E-learning Course on Pediatric Cardiology Basics

**DOI:** 10.2196/mededu.5434

**Published:** 2017-05-10

**Authors:** Ana Cristina Oliveira, Sandra Mattos, Miguel Coimbra

**Affiliations:** ^1^ Faculdade de Ciências da Universidade do Porto and Faculdade de Medicina da Universidade do Porto Porto Portugal; ^2^ Círculo do Coração Unidade de Cardiologia e Medicina Fetal Real Hospital Português de Pernambuco Recife Brazil; ^3^ Instituto de Telecomunicações Faculty of Sciences of the University of Porto Porto Portugal

**Keywords:** distance learning, continuing medical education, pediatrics, cardiology, congenital heart defects

## Abstract

**Background:**

Early detection of congenital heart disease is a worldwide problem. This is more critical in developing countries, where shortage of professional specialists and structural health care problems are a constant. E-learning has the potential to improve capacity, by overcoming distance barriers and by its ability to adapt to the reduced time of health professionals.

**Objective:**

The study aimed to develop an e-learning pediatric cardiology basics course and evaluate its pedagogical impact and user satisfaction.

**Methods:**

The sample consisted of 62 health professionals, including doctors, nurses, and medical students, from 20 hospitals linked via a telemedicine network in Northeast Brazil. The course was developed using Moodle (Modular Object Oriented Dynamic Learning Environment; Moodle Pty Ltd, Perth, Australia) and contents adapted from a book on this topic. Pedagogical impact evaluation used a pre and posttest approach. User satisfaction was evaluated using Wang’s questionnaire.

**Results:**

Pedagogical impact results revealed differences in knowledge assessment before and after the course (Z=−4.788; P<.001). Questionnaire results indicated high satisfaction values (Mean=87%; SD=12%; minimum=67%; maximum=100%). Course adherence was high (79%); however, the withdrawal exhibited a value of 39%, with the highest rate in the early chapters. Knowledge gain revealed significant differences according to the profession (X22=8.6; P=.01) and specialty (X22=8.4; P=.04). Time dedication to the course was significantly different between specialties (X22=8.2; P=.04).

**Conclusions:**

The main contributions of this study are the creation of an asynchronous e-learning course on Moodle and the evaluation of its impact, confirming that e-learning is a viable tool to improve training in neonatal congenital heart diseases.

## Introduction

### Background

Managing congenital heart diseases is a worldwide problem [1]. Early detection through newborn screening can potentially improve the outcome of these diseases [2]. In newborns, congenital heart diseases can be detected by auscultation, pulse oximetry, radiography, catheterization, although transthoracic echocardiogram, a specialized form of ultrasound, is the elected exam for diagnosis [3,4]. Pediatric cardiologists usually perform this examination, but in developing countries there is shortage of professional specialists, which are often concentrated in larger urban centers, hindering the widespread population screening and causing a need for constant transferal of patients from the isolated regions to reference health centers [5].

### Context

In response to these challenges, the Health Secretary of Paraíba, in Brazil established a pediatric cardiology network [6]—Rede de Cardiologia Pediátrica (RCP)—in partnership with Círculo do Coração *,* a civil nonprofit organization from Recife, in order to create a neonatal screening program for the whole state of Paraíba and a hospital facility designed to manage patients. Of the 20 maternity centers covered by this network, only 7 had neonatal ICU beds. The remaining are level-1 district centers. In only one center, in the capital city of Joao Pessoa, there is a pediatric cardiologist available for echo and clinical diagnosis. No center performs cardiac surgery. This network provides perinatal and neonatal care in remote areas supervised by telemedicine; it was created due to the need to train local physicians and involve local professionals on screening, diagnosis, therapeutic treatment and management of congenital heart diseases in fetuses, newborns, and children of the public health system.

Echocardiography is used to diagnose congenital heart disease when either there is an abnormal clinical examination or an abnormal pulse oximetry. Abnormal pulse oximetry results are automatically noted on a database of the network, allowing the network to contact the clinic and request that they follow up any babies with abnormal test results. These active search protocols track the discharged neonates and ensure that abnormal findings are acted on.

Echo image acquisition and its interpretation is a combined process developed within this Web-based telemedicine network where trainees are constantly supervised by cardiologists. Not only the whole process is documented in the Web database system to track the apprentice's development, but also whenever needed, live interaction is called to adjust practical aspects. Initially, all echocardiograms needed to be performed with Web-based supervision by the pediatric cardiologist, as part of the neonatologists’ training, but with time cardiologists would only be requested for direct Web-based supervision when pathological findings were suspected. As there are always new neonatologists being trained, this learning process and interaction between teams is a continuous cycle [7].

### E-Learning for Health Care

With the increasing use of Internet information and communication technologies, e-learning has emerged as a widely accepted modality in medical education [8]. For its convenience and its potential for cost savings, it has become popular among the medical education community [9]. E-learning is known to offer learning opportunities where there is limited access to teaching in a specific field, either because of a lack of qualified or geographically distant teaching institution [10]. Therefore, e-learning can be a powerful tool to increase the capacity of health professionals in constricted contexts for neonatal echocardiography congenital heart disease screening. There is evidence in the literature that e-learning is a useful tool for overcoming barriers to health professionals training [10]. A review carried out by Frehywot et al on e-learning in medical education in resource-constrained LMICs suggests that e-learning may be effective for increasing capacity in rural settings, although evidence is still limited.

The convenience, the self-paced and learner-centered learning, and the creation of a global learning community, are some significant benefits of e-learning that have been discussed in many articles [8,10-24].

In medical contexts, e-learning program results such as efficiency and costeffectiveness have typically gone unreported [25]. In nonmedical contexts, there is evidence that e-learning can result in cost savings of up to 50% over traditional learning programs, due to reduced instructor training time, travel and labor costs, institutional infrastructure, and the possibility of expanding programs with new technologies [8,9,26].

Nowadays, there is an increasing demand for e-learning courses [27]. Many software platforms and learning management systems (LMS) are being used to support Web-based courses for online continuing medical education. Among the various LMS, there is Moodle (Modular Object Oriented Dynamic Learning Environment; Moodle Pty Ltd, Perth, Australia), a well-known platform that is considered one of the best open-source LMS, in what concerns user-friendliness and adaptivity [28]. Moodle allows the integration of a broad range of educational resources, activity modules, such as Forums, Wikis, and Databases, that build a rich collaborative community of learning around a subject matter depending on the learning goal. Moodle can also be used to deliver content to students (such as standard SCORM packages) and for learning assessment; it provides assignments or quizzes. As another advantage, its interface allows surfing through the contents intuitively [28,29]. The reason we decided to use Moodle in this study is because we had access to it in the Faculty of Medicine of the University of Porto that could host the Moodle course, and given its utility, we did not need to address the costly development of a new platform.

### Objectives of the Study

In this context, as primary outcomes we aim to (1) develop an e-learning Pediatric Cardiology Basics Moodle course for nonspecialists and (2) evaluate its pedagogical impact and user satisfaction.

As secondary outcomes, we want to understand whether there are significant differences between different types of professionals undergoing the course and to measure the adherence to the course in this specific scenario.

## Methods

### The E-Learning Course Description

We chose to build a new course, as the few actual existing options are in English, and also associated with other institutions. The course was in Brazilian Portuguese, implemented on Moodle, hosted on the server at the Faculty of Medicine of the University of Porto, Portugal. The contents of the course were mainly based on the “Cardiologia para o Pediatra” book [30], which was used in this network to teach their professionals, and was written by a specialist Dra Sandra Mattos, stakeholder of the RCP network. This book follows a didactic approach, where the basic concepts for neonatal screening of congenital heart diseases are addressed, such as the current standard clinical protocols and guidelines to apply [31,32]; proposed by the American Association of Cardiology and the Association for European Paediatric and Congenital Cardiology, concepts of cardiac neonatal anatomy and physiology, how to obtain the ultrasound images, and for each anatomic window, which echocardiographic findings to expect and look for. The course was structured into 3 modules, with a total of 8 chapters (Table 1).

**Figure 1:**
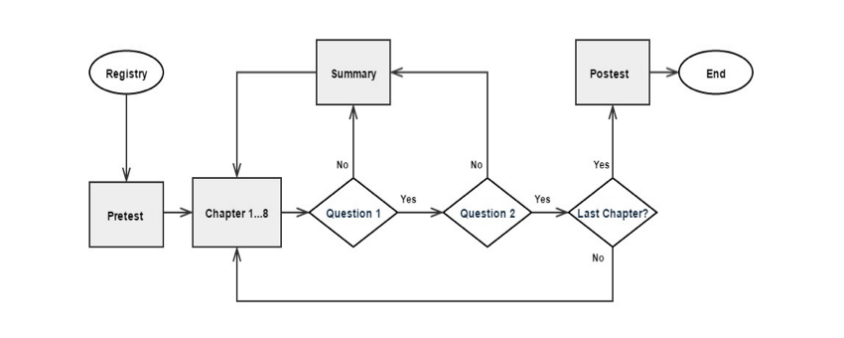
Course flow diagram.

The content was presented using text, images, and videos. Diagnostic images and videos were collected directly from the RCP network at the Real Hospital Português in Recife, Brazil. A total of 22 images and 36 videos were used in this course, which were anonymized and illustrated examples of planes and imaging cuts, without any association with identifiable data from patients. Pediatric cardiology specialists from the cardiology and fetal medicine unit at Real Hospital Português reviewed all contents. Screenshots are presented in Figure 1.

**Table 1:** Description of the main contents contained in the intervention e-learning course.

Modules	Chapters	Contents
Foreknowledge	Cardiac neonatal anatomy Physiology of neonatal circulation Physical ultrasound principles	Internal configuration of the heart Fetal circulation Neonatal circulatory changes The ultrasound properties Transducers Echocardiography
Echocardiogram screening	How to obtain the ultrasound images Different echocardiographic modalities	4-chamber image Left ventricle outflow track image Right ventricle outflow track image Pathologies to exclude in each image Bidimensional mode M mode Doppler mode
Pathologies	Identified pathologies in the 4-chamber image Identified pathologies in the outflow tracts image Difficult to diagnose pathologies images in the neonatal period	Interventricular communication Defect of atrioventricular septum Tricuspid atresia Ductus arteriosus Aortic stenosis Pulmonary stenosis Tetralogy of Fallot Transposition of the great vessels Truncus arteriosus Interatrial communication Coarctation of the aorta Total anomalous pulmonary venous drainage

### Recruitment and Implementation Strategy

Participants were recruited via an email disclosure within the RCP network platform with a brief description of the study, the link to the course, a tutorial for Moodle log, and the password for enrolment in the course. To access it, the learners should register on the Moodle website according to their personal information. Thus, website security was guaranteed through an authentication mechanism with username and password. After that, we had access to the participant’s personal email given at the registry, and then we could do the follow-up of each participant by email.

The course was created and revised between April 2014 and July 2014. We contacted the participants in September 2014, and those who registered to do the course first did the pretest and then had access to the asynchronous course, which was given until November 2014.

**Figure 2:**
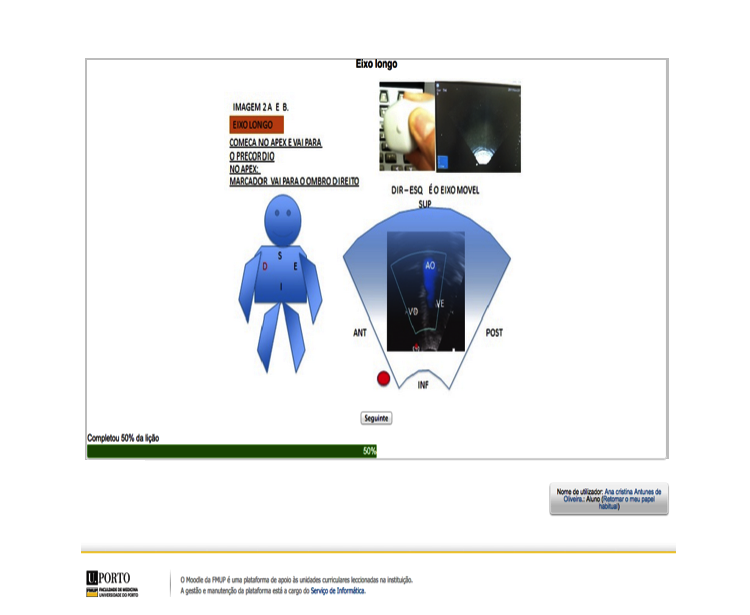
Screenshot of one of the echocardiographic views and schemes (left), and of one of the self-assessment tests (right) included in the e-learning course.

The course was built in a unidirectional way (Figure 2). First, the participants registered on the Moodle website. Then, a pretest of 16 questions was available during 20 min. After the answers submission, the first chapter was available. At the end of each chapter, a summary with the main key-points and a formative test of two multiple-choice questions for self-assessment was presented. These intermediate tests allowed immediate knowledge self-assessment. The next chapter would only be available if the user had given the correct answers. In case of error, the learner would be directed to a summary of the lecture, and then it was possible to go back to the previous lecture or repeat the assessment. At the end of these 8 chapters, a final summative assessment of 16 questions corresponding to the posttest was available during 20 min. After the final approval, a certificate was sent to the learners.

The learning activities chosen were lessons that corresponded to each chapter. We organized the content, images, and videos in different pages as it corresponds to different topics. The learner could control the lecture flow by pressing control buttons located at the end of that interface, moving forward or backwards, allowing the lecture to flow from the beginning to the end, page by page, managing his own learning process.

As the course was performed asynchronously, the learner could observe his evolution learning at any time during the course through a progress status bar that updates dynamically in order to manage his self-learning.

### Evaluation Strategy

An important method of assessing educational training is a framework developed by Kirkpatrick [33,34], which focuses on 4 levels: reaction, learning, behavior, and results.

Numerous studies [16,21,35-42] have been carried out to assess the benefits of e-learning in medical education. Very often, evaluative education studies [38,43] rely solely on the reaction level and learning over the behavior and results levels [16,44,45].

#### Pre- and Postintervention Test

Concerning the learning assessment, the most common method described in literature is the pre- and posttesting self-controlled method, with multiple-choice test scores.

In this study, the participants were asked to do a pretest before taking the course and a posttest after completing the course. We used the same questions for pre and posttesting, so that we can guarantee the same level of difficulty and comparable results [46]. The test was structured with 16 different multiple-choice questions. They were all single-select questions and each question had 2-5 response options. The assessment questions were text-based to test knowledge based on the key ideas, learning outcomes, and objectives established for the course. In this test, a total of 2 questions were related to each of the course’s 8 modules (Table 1), which were also the same end-of-chapter practice questions. The participants could only proceed to the next chapter after answering these end-of-chapter practice questions correctly. An expert on pediatric cardiology revised the questions making sure they were appropriate to the course content. To improve the authenticity of the answers by discouraging access to support materials, participants had only 20 min to complete the 16-question test, so a time controller and a progress bar were available. After that time, if they had not submitted, the answers would be automatically saved. At the end, the participants could see the test result, but not the correction, and they could not repeat the test. The technology we used to provide this evaluation tool was the Moodle lesson questions, a free Web-based office suite and data storage service.

#### User’s Satisfaction Questionnaire

The reaction assessment is mostly done by questionnaires. One of the most cited questionnaires for assessing user satisfaction was developed by Wang [47]. Wang created an e-learning satisfaction model that consists of 26 items related to 4 qualities: content quality, learning interface quality, personalization quality, and learning community quality. However, the last 2 questions refer to global measurement in the context of end-user satisfaction, first developed by Doll in 1988 [48], and related to overall satisfaction and overall success. The measurement scale used was a 7-point Likert-type scale, with anchors ranging from “strongly disagree” to “strongly agree.” Globally, this questionnaire was shown to have a reliability (Cronbach alpha) of .95 [47].

For our study, we used the questions related to content quality, learning interface quality, and personalization quality from Wang’s questionnaire [47]. We did not use questions related to learning interaction quality, because the developed e-learning course was asynchronous. We used a Portuguese translated existing version from Wang’s questionnaire [49]. The technology used to apply this evaluation instrument was the Moodle survey module. The satisfaction questionnaire became available to the participants who had taken the course after they did the postcourse.

### Statistical Analysis

The purpose of the data analysis was to determine whether there was a significant difference between the test score before and after the course. We considered learning improvement as the ratio of the difference between scores and the preintervention score. The learning efficiency is the learning improvement per hour of the course.

Data analysis was performed by descriptive and inferential statistics, using the IBM SPSS Statistics software version 22.0.

According to the fulfillment of the criteria necessary to perform parametric hypothesis testing, it was concluded that the sample did not follow a normal distribution. Thus, we used the following nonparametric tests: Wilcoxon Signed-Rank test, Mann-Whitney *U* Test and Kruskall-Wallis *H* test. For all this statistical analysis, we considered a significance level of 5% [50].

## Results

### Summary

**Figure 3:**
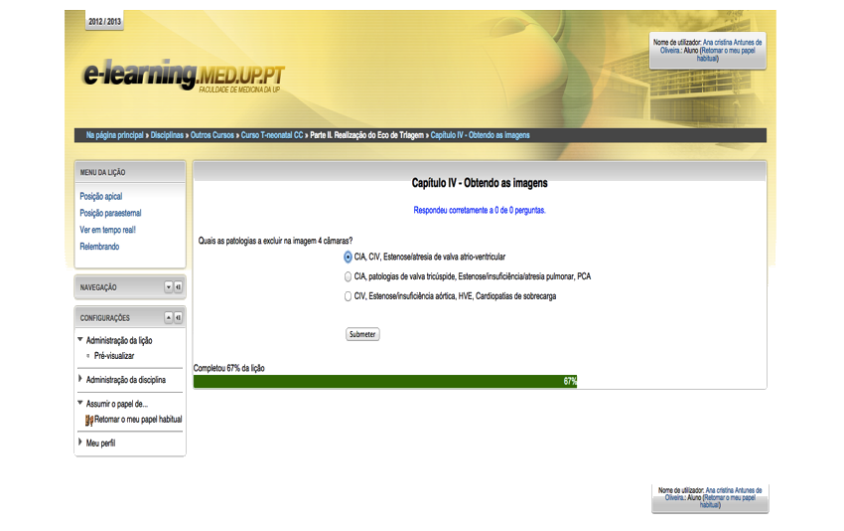
Screenshot of one of the self-assessment tests included in the e-learning course.

**Figure 4:**
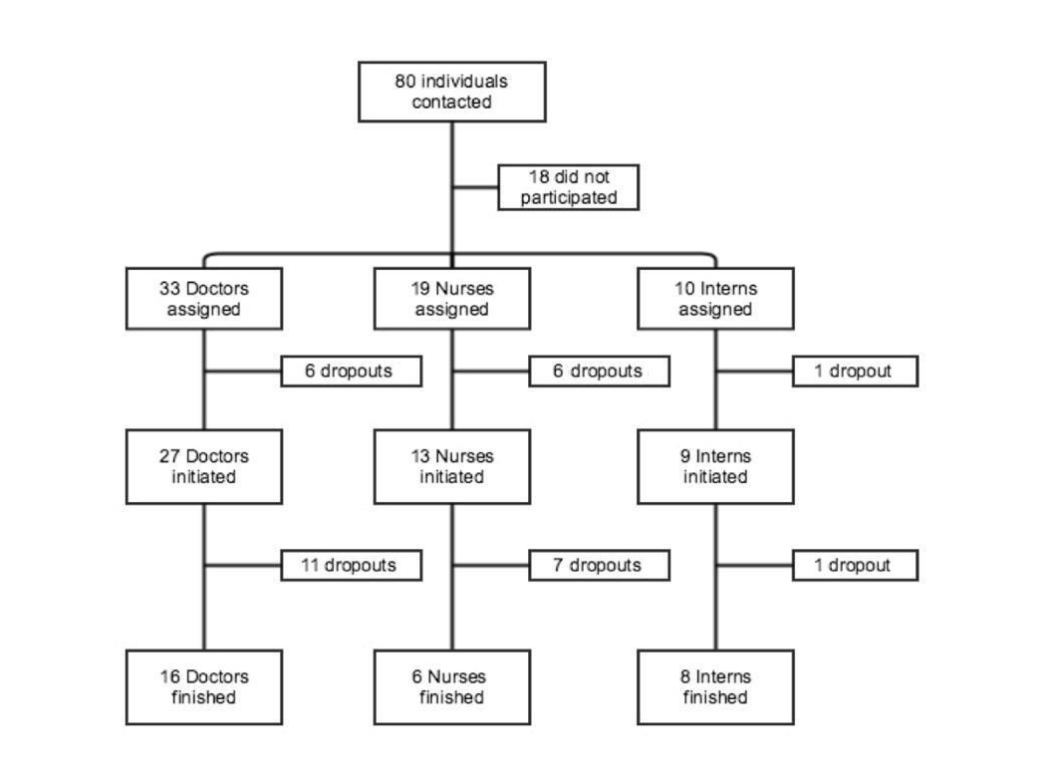
Participant flow diagram showing the enrolled sample and respective dropouts.

**Figure 5:**
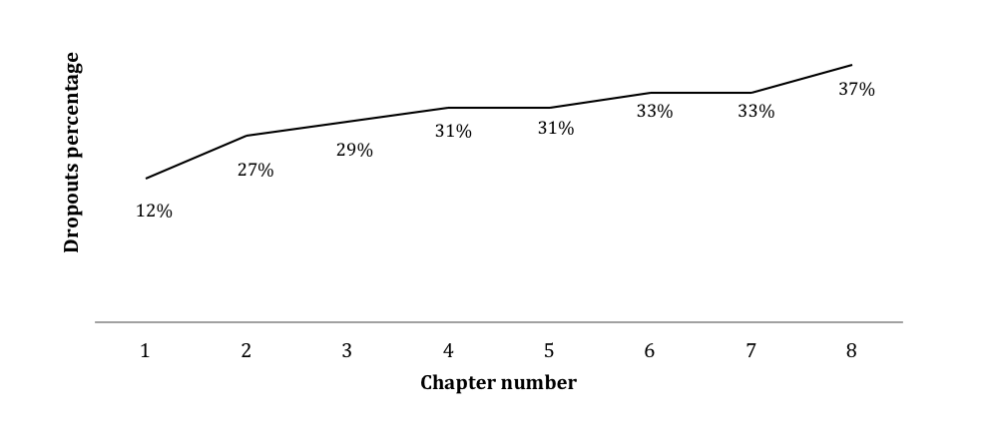
Percentage of participants who dropped by chapter.

The target population in this study was the health care professionals—neonatologists, pediatricians, obstetricians, nurses, and internship medical students—who work in the RCP network. This convenience universe potentially includes a total of around 80 people. We obtained 78% (62/80) registrations. Concerning adherence (Figure 3), 79% (49/62) started the course. At the end, 61% of the participants (30/49) managed to complete the course and 39% (19/49) dropped out (Figure 4). See the percentage of participants who dropped by chapter (Figure 5). From the participants who had taken the course, 67% (20/30) responded to the satisfaction questionnaire.

### Sample Description

The total sample consisted of 62 registered health care practitioners, including 49 female (79%) and 13 male elements (21%); 49 individuals started the course, including 39 female (80%) and 10 male elements (20%); and 30 participants completed the course, including 22 female (73%) and 8 male elements (27%; Table 2).

With regard to the registered participants, most are doctors (n=33; 53%) and mostly are of Neonatology (n=24; 39%) and Pediatrics (n=23; 37%) departments. This ratio remains the same for those participants who initiated the course (Table 3).

Regarding the state where they practice, 45 (73%) works in Paraíba and the other 17 (27%) in Pernambuco.

### Pedagogical Impact

Regarding the pretest, 67% (n=20) passed (test score≥50%) and 33% (n=10) of participants failed (test score<50%). The test scores ranged between 0 and 100%. However, none of the participants failed to complete all the test items within the time limit. With respect to the posttest, 100% (n=30) of the participants passed.

The differences between the test scores before and after the course were all positive. There were no negative differences or equal scores before and after the course. A Wilcoxon signed-ranks test indicated that the median [Mdn (P25-P75)=94 (81-100)] posttest ranks were statistically significantly higher than the median [Mdn (P25-P75)=56 (38-69)] pretest ranks (*Z*=-4.788; *P*<.001).

Doing an intent-to-treat analysis by comparing the mean ranks (Mann-Whitney–U Test) of the score from the participants who did not do the course [Mdn (P25-P75)=50 (29-65); n=19] with those who did the course [Mdn (P25-P75)=94 (81-100); n=30], we found that test scores in those who did the course were statistically significantly higher than those who did not (*U*=21.500; *P*<.001).

**Figure 6:**
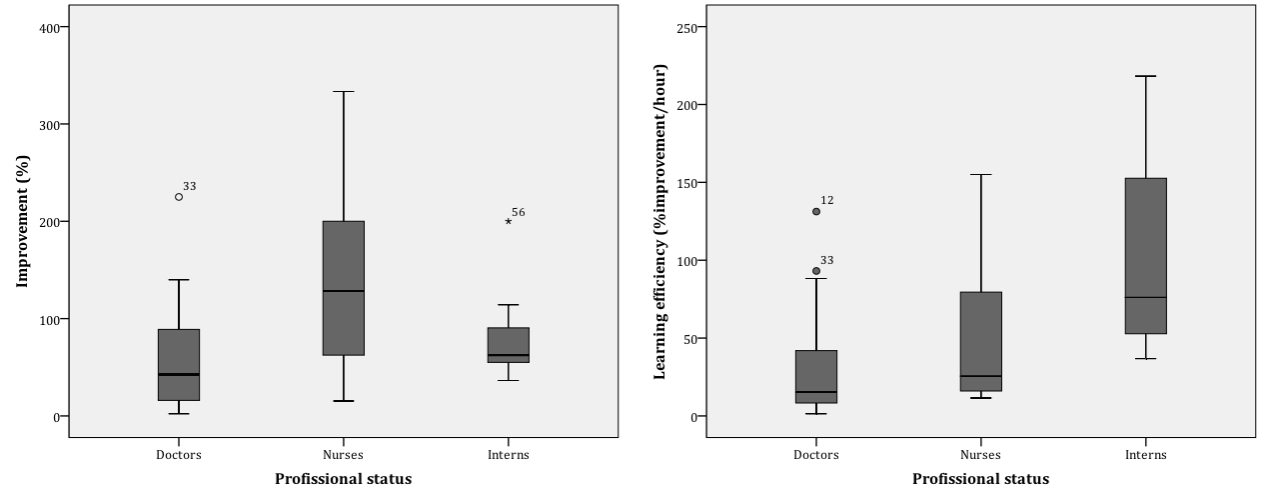
Improvement (left) and learning efficiency (right) stratified by professional status.

Globally, the difference between the final and the initial scores can indicate the learning impact of the course [Mdn (P25-P75)=34 (19-50); n=30]. Moreover, the improvement in the results was related to what the participants already knew before the course [Mdn (P25-P75)=61 (36-114); n=30]. The efficiency was how much they improve per hour dedicated to the course [Mdn (P25-P75)=31 (12-80); n=30] (Figure 6). The maximum time dedicated to the course was 09 h 58 min and the minimum 25 min [Mdn (P25-P75)=01:47 (01:08-03:01); n=30].

We found the initial scores were statistically significantly different (X^2^_2_=13.8 *P*=.001) between professions (Table 4). Although nurses hadn’t the highest final score, they were the ones who exhibited the highest difference between the scores, more than doubling it, but with less efficiency, as they also dedicated more time to the course. However, internship medical students had the highest learning efficiency, improving their knowledge per hour, as they dedicated much less time to the course than doctors and nurses. Predictably, doctors have lesser benefits from such an e-learning course. There were significant statistical differences in the improvement between professions (X^2^_2_=8.6; *P*=.01).

**Table 2:** Frequency and percentage of participants by gender.

Participants	Female, n (%)	Male, n (%)
Total sample (n=62)	49 (79)	13 (21)
Started the course (n=49)	39 (80)	10 (20)
Completed the course (n=30)	22 (73)	8(27)

**Table 3:** Frequency and percentage of participants by profession and specialty.

Participants		Assigned (n=62), n (%)	Initiated (n=49), n (%)	Concluded (n=30), n (%)
**Profession**				
	Doctors	33 (53)	27 (55)	16 (53)
	Internship medical students	10 (16)	9 (18)	8 (27)
	Nurses	19 (31)	13 (27)	6 (20)
**Speciality**				
	Neonatology	24 (39)	19 (39)	18 (60)
	Obstetrics	6 (10)	5 (10)	4 (13)
	Pediatric Cardiology	9 (14)	8 (16)	3 (10)
	Pediatrics	23 (37)	17 (35)	5 (17)

**Table 4:** Median and percentile Tukey’s hinges (P25-P75) of the scores in study by profession.

Indicators	Doctors (n=16)	Internship medical students (n=8)	Nurses (n=6)	*P* value^a^
Initial score	63 (53-78)	50 (41-59)	40 (31-50)	.001
Final score	99 (88-100)	94 (81-94)	88 (81-100)	.24
Difference^b^	27 (13-44)	34 (28-44)	51 (31-63)	.13
Improvement (%)^c^	43 (16-89)	63 (55-91)	128 (63-200)	.09
Efficiency (%)/h^d^	15 (8-42)	76 (53-153)	26 (16-80)	.01
Dedication (hh:mm)	02:01 (01:32-03:06)	00:50 (00:37-01:06)	03:07 (02:09-03:54)	.45

^a^Kruskal-Wallis *H* test.

^b^Final score - initial score.

^c^([final score - initial score]/ initial score)*100.

^d^([final Score - Initial score]/ initial score)*100)/hour.

**Figure 7:**
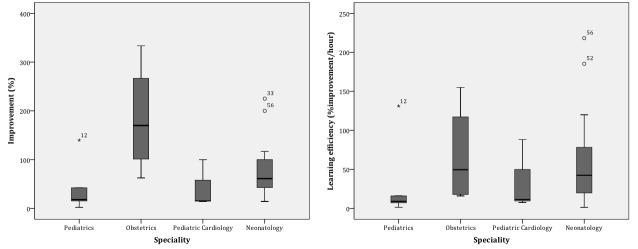
Improvement (left) and learning efficiency (right) stratified by specialty.

Concerning specialty (Table 5), the obstetricians were the ones who benefited the most, with the highest difference between the scores, the best improvement and efficiency (Figure 7), and were the most dedicated to the course. There were statistically significant differences in the improvement (X^2^_2_=8.4; *P*=.04) and in the course dedication (X^2^_2_=8.2; *P*=.04) between the different specialties.

**Table 5:** Median and percentile Tukey’s hinges (P25-P75) of the scores in study by specialty.

Indicators	Neonatology (N=18)	Obstetrics (N=4)	Pediatric Cardiology (N=3)	Pediatrics (N=5)	*P* value^a^
Initial score	56 (44-63)	37 (25-46)	87 (68-87)	69 (69-81)	.06
Final score	91 (81-100)	88 (81-87)	100	98 (94-100)	.15
Difference^b^	38 (25-44)	60 (45-63)	13 (13-32)	13 (13-29)	.06
Improvement (%)^c^	61 (43-100)	170 (101-267)	15 (15-58)	18 (15-42)	.039
Efficiency (%)/h^d^	42 (20-78)	50 (18-117)	12 (10-50)	9 (7-16)	.28
Dedication (hh:mm)	01:39 (00:54-03:01)	03:12 (02:20-05:30)	01:20 (01:14-1:36)	01:35 (01:08-2:08)	.04

^a^Kruskal-Wallis *H* test.

^b^Final score-initial score.

^c^([final score-initial score]/ initial score)*100.

^d^([final score-initial score]/ initial score)*100)/hour.

### User Satisfaction

Only 20 (67%), out of the 30 participants who completed the course answered the satisfaction questionnaire. This was disappointing, showing that it was simpler to motivate these professionals to gain knowledge, than to contribute to a research study.

Globally, the satisfaction with the e-learning course was positive (μ=87%; σ=12%; minimum=67%; maximum=100%), whereas 6 (30%) learners were totally satisfied with the e-learning course. Regarding global measures (Q14 and Q15), 17 (88%) were satisfied and considered that the e-learning course was successful (6 and 7 points in the 7-point Likert-type scale). Considering content quality (Q1-Q4), 17 (88%) learners think that this course fits their needs, is useful, sufficient, and up to date.

About the learning interface quality (Q5-Q9), 15 (76%) learners found the e-learning course user-friendly, stable, making it easy to find contents needed. Concerning personalization quality (Q10-Q13), 16 (84%) participants think the e-learning course enables them to control the learning progress, to learn the content needed, to choose what to learn and record their learning progress and performance.

Within these 20 participants, all claim the necessity of continuing medical education (CME) and 18 (90%) said that they would like to do CME by e-learning systems, whereas the other 2 (10%) said that maybe they will be interested in CME by e-learning.

## Discussion

### Principal Findings

The first main result of this study was the development of an e-learning course for the neonatal screening of congenital heart diseases. Using a free open-access tool such as Moodle and adapting the pedagogical contents of a well-established book for teaching this subject did not require advanced programming skills and led to an effective e-learning course. Given our successful results, it is expectable that future e-learning courses that specifically use best practices for multimedia learning should have an even stronger impact.

The second principal outcome was the statistically significant results obtained in the used metrics of pedagogical impact, with quite interesting proportions of learning with just a few hours of training. These results meet the general literature reviews that identified e-learning as superior to noneducation intervention [45], or as having similar effects to traditional learning [51], and more effective when combined with traditional learning [29] (b-learning). Although, in some contexts, e-learning may not be an alternative for the traditional face-to-face learning method, it can always offer a contribution and be a complement, and a useful adjunct to traditional education [51,52].

When comparing professions, we confirmed that the background knowledge about pediatric cardiology varies by professional group. By the end of the course nurses were the ones who learned more, although they did not score the highest final score. The heterogeneity of time spent in learning was also perceived. Internship medical students had the highest learning efficiency, dedicating much less time to the course than doctors and nurses. Predictably, doctors have lesser benefits from such an e-learning course.

Concerning specialty, the obstetricians were the ones who improved their knowledge the most, with the highest difference between the scores, the best improvement and efficiency, and were the most dedicated to the course. There were statistically significant differences in the improvement and in the course dedication between the different specialties, in this case confirming that more time dedicated to the course does translate into higher knowledge gain.

The third principal result was the encouraging assessment of the user satisfaction questionnaire. However, a third of the students who completed the course did not complete this questionnaire. This might be an important consideration for future studies, with regard to the interest and motivation of the participants to answer satisfaction questionnaires.

Regarding the adherence to this study, although the number of participants can appear low, we must understand the context in which this course was applied. From a total of around 80 people who are fully engaged in their daily health care routine and who voluntarily accepted to participate and complete an 8-lesson course in their free time, we consider that 30 is a successful result that reflects the need and enthusiasm generated by the proposed initiative.

### Limitations

Relevant limitations include the small sample size that affects the generalization of the results, and the limited implementation time, as the learners have their own agenda and priorities.

For the analysis, we had to use nonparametric tests because some of the variables did not follow a normal distribution and mainly because of the sample size. It would be definitely more interesting to use parametric methods to prove that there were significant differences before and after the course and the interactions between professions and specialty in a more robust manner, but for that possibility we would have to have a greater number of participants.

In addition, we have the impact of the exposure to the pretest and the end-of-chapter practice questions, which were identical to the ones that were used in the posttest. Although there was an expert validation of the test questions used before and after the course, the test was not assessed for its reliability or validity metrics.

The validity of the satisfaction assessment should be carefully considered, because we used a nonvalidated translation to a Portuguese version of the user satisfaction questionnaire, as it was not possible to find in the literature a Portuguese validated one for e-learning systems.

Another limitation of this work is the fact that the socioprofessional questionnaire was applied only at the end of the intervention. This situation limits our information about the description of our sample and other factors that could influence the adherence and learning process.

Concerning the evaluation strategy, we also faced the risk of a slight bias because we did not control if learners resorted to external sources in order to provide correct answers to the tests.

### Conclusions

Globally, this study highlights the importance of training neonatologists and other health care professionals in the neonatal care units to screen for congenital heart disease. We consider the high rate of participation an important aspect of our study (78%), which reflects the great interest shown by these professionals to promote their professional skills. They took advantage of this learning opportunity, which confirms that these health care professionals are committed to responding to new challenges and evolving paradigms.

This study contributes to the Brazilian continuing training programs, as we did not find any similar course related to neonatal screening of congenital heart disease. It would be interesting to conduct additional assessments to demonstrate effective consolidation of knowledge gain. For future work, we also intend to assess the remaining levels of the Kirkpatrick framework, “behavior” and “results,” with respect to change in neonatal screening behavior and improved congenital heart disease detection. To do so, we plan to measure the number of telemedicine consultations conducted by the participants after the course and the number of congenital heart disease detected by them.

Our global results show that e-learning can provide statistically relevant knowledge gains in health care professionals in a neonatal screening context. We believe that this study underlines the importance of e-learning as a viable technology for training, especially in impoverished contexts. E-learning should be considered for continuing medical education in low- and middle-income countries, not only due to budget constraints, but also due to resource-constrained environments.

## Abbreviations

CME: continuing medical education
LMS: learning management system
Moodle: Modular Object Oriented Dynamic Learning Environment
